# Review of Prospects of Biological Fluid Biomarkers in Osteoarthritis

**DOI:** 10.3390/ijms18030601

**Published:** 2017-03-12

**Authors:** Lich Thi Nguyen, Ashish Ranjan Sharma, Chiranjib Chakraborty, Balaji Saibaba, Moo-Eob Ahn, Sang-Soo Lee

**Affiliations:** 1 Institute for Skeletal Aging & Orthopedic Surgery, Hallym University-Chuncheon Sacred Heart Hospital, Chuncheon 24252, Korea; lichbio@gmail.com (L.T.N.); boneresearch@hallym.ac.kr (A.R.S.); drchiranjib@yahoo.com (C.C.); balajijipmer@gmail.com (B.S.); 2 Department of Bio-Informatics, School of Computer and Information Sciences, Galgotias University, Greater Noida 203201, India; 3 Department of Emergency Medicine, Hallym University-Chuncheon Sacred Heart Hospital, Chuncheon 24252, Korea

**Keywords:** osteoarthritis (OA), biomarker, marker of joint metabolism, inflammatory marker, genetic marker

## Abstract

Osteoarthritis (OA) is a degenerative disease of the joints and is one of the leading causes of disability in adults. However, there are no key therapeutics for OA and medical treatment is based on managing the symptoms and slowing down progression of the disease. Diagnostics based on clinical examination and radiography have provided little information about metabolic changes in joint tissues, disease onset and progression. Due to lack of effective methods for early detection and evaluation of treatment outcome, the measurement of biochemical markers (biomarkers) shows promise as a prospective method aiding in disease monitoring. OA biomarkers that are present in biological fluids such as blood, urine and synovial fluid, sources that are easily isolated from body, are of particular interest. Moreover, there are increasingly more studies identifying and developing new biomarkers for OA. In this review, efforts have been made to summarize the biomarkers that have been reported in recent studies on patients. We also tried to classify biomarkers according to tissue metabolism (bone, cartilage and synovial metabolism markers), pathological pathways (inflammatory and genetic markers) and biological function (chemokines, growth factors, acute phase proteins, etc.).

## 1. Introduction

Osteoarthritis (OA) is the most common adult joint disease, affecting people all over the world. The disease frequently occurs in the hands, knees, hips and spine and is associated with symptoms of inflammation, stiffness and loss of mobility. Despite OA being well-known as a consequence of cartilage degradation, damage to subchondral bone, synovium, capsule, periarticular muscles, sensory nerve endings and meniscus also contribute to the etiology and progression of OA [1]. OA is characterized by progressive degradation of articular cartilage and remodeling of subchondral bone with formation of osteophytes [2]. This disease has been described as having an association with sex and age. There is increased frequency of severe OA in those over 50 years of age, and the incidence of OA is higher in women than in men [3]. Estrogen decline in older women is known as a main factor in cartilage degradation that leads to OA [4]. Moreover, other factors, such as genetics, obesity and overuse of joints, are also known contributors to the risk of developing OA [1,5].

**Figure 1:**
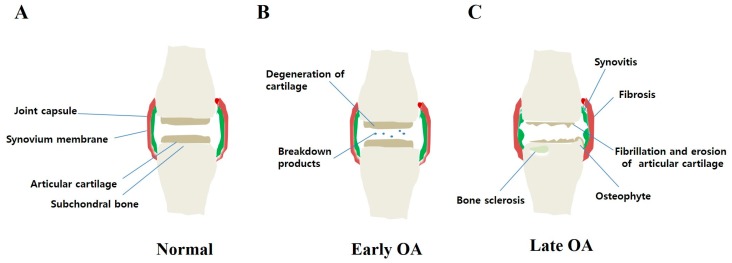
Model of pathologic progression of osteoarthritis (OA). OA is a slow, progressive disease. (**A**) Normal joint without any damages; (**B**) Early OA is always difficult to detect, characterized by cartilage degeneration and release of breakdown products into the synovial fluid environment; (**C**) Late OA is an obvious event, with cartilage loss (fibrillation and erosion of articular cartilage) and osteophyte formation. Damage of the subchondral bone, synovium and capsule may also occur (bone sclerosis, synovitis, and fibrosis, respectively).

A pathologic progression of OA can be described generally by three stages. Stage I is characterized by the proteolytic breakdown of cartilage matrix, which results from the disruption of chondrocyte metabolism leading to increased secretion of degradation enzymes such as collagenases and aggrecanases. Stage II involves the fibrillation and erosion of the cartilage surface, followed by a release of breakdown products (proteoglycan and collagen fragments) into the synovial fluid. In stage III, synovial inflammation occurs when breakdown products are phagocytized by synovial cells, leading to production of inflammatory cytokines and proteases. Finally, these molecules, in turn, enhance a more comparable catabolic effect on chondrocyte metabolism, inducing degradative proteases and decreasing proteoglycan and collagen synthesis and, therefore, accelerating progression of the disease (vicious cycle) (Figure 1).

The driver of OA is still a question. The most popular theory suggests that OA is initiated by disorder of chondrocytes metabolism and cartilage degradation. An “inflammatory” theory, otherwise, suggests that synovitis is the primary trigger of the OA process, and it results in cartilage damage [6]. Moreover, a recent evidence even suggests that subchondral bone may have a role in OA onset as it showed that aberrant bone formation may be responsible for degeneration of articular cartilage [7]. Taken together, OA is a complex disease and cartilage, synovium or subchondral bone could become a driver for it.

The etiology of OA is diverse and treatments based on therapeutics to preserve the joint and total joint replacement are an economic burden, especially when the disease becomes severe. Therefore, early detection is important to cease or slow down the process of the disease. Additionally, while OA is a chronic and slowly progressive disease, detection for a therapeutic response requires rapid indicators (with strong predictive potential for disease diagnosis and progression). Diagnosis and detection are currently based on clinical symptoms in combination with radiography, which is relatively insensitive and occurs when the disease is already in late phases. Radiography has been used to visualize the features known as the pathologic features of late progression of OA such as bone sclerosis, subchondral sclerosis, osteophytes and joint space narrowing (JSN)—an indirect sign that reflects cartilage loss. This method has limitations; in some cases, the joint damage is associated with other tissues such as cartilage, synovium, meniscus, ligaments, etc. Magnetic resonance imaging (MRI) and ultrasound overcome the drawback of radiographic imaging [8]. In case of MRI, it enables visualization of various types of damage that occur in joint, for example, cartilage lesions, cartilage thickness loss, bone marrow lesions (BMLs) and meniscal tear. According to a recent study of Ramonda et al., synovitis and BMLs detected by MRI were associated with pain, an early progression feature of erosive hand OA [9]. Even though MRI provides a diagnostic approach aiding early detection of OA, this method cannot become popular due to its high cost. Ultrasound (US) is a useful technique which enables visualization of articular soft tissue structures, however, it is limited to visualizing the entire joint due to acoustic shadowing [10]. Besides, detection based on molecular markers is not only an easy and less costly method but also can provide quantitative, reliable and early detection of OA, therefore, it is considered as a prospective method for management of this disease.

Hence, the aim of this review is to summarize the investigation and development of common molecular markers for OA with the limitation of using markers obtainable in biological fluids.

## 2. Biomarkers for Cartilage, Bone and Synovium Metabolism

### 2.1. Markers of Cartilage Metabolism

Type II collagen is a major component of the cartilage matrix and its synthesis and breakdown are closely related to cartilage metabolism. Many studies have focused on synthesis and degradation of type II collagen to identify biochemical markers for OA. Generally, type II collagen is synthesized as procollagen molecules including the procollagen type II N-terminal propeptide (PIINP) and the procollagen type II C-terminal propeptide (PIICP). During maturation, the propeptides are cleaved off and released into biological fluids. Therefore, the levels of these peptides reflect type II collagen synthesis. It has been shown that PIICP concentrations in joint fluid are a prognostic marker for early OA in the knee as the level of PIICP was found to correlate with risk factors such as obesity and varus alignment [11] (Table 1).

Moreover, type II procollagen is produced in two forms (procollagen type IIA N-terminal propeptide, PIIANP and procollagen type IIB N-terminal propeptide, PIIBNP); different in the N-terminal) as the result of alternative RNA splicing. A decrease in serum PIIANP has been observed in patients with knee OA and rheumatoid arthritis (RA) [12,13]. A study by Sharif et al. investigated serum PIIANP levels in patients with mild-to-moderate knee OA for a period of 5 years and showed that disease progression correlates with higher levels of serum PIIANP, and patients within the highest quartile of PIIANP levels have the highest risk of OA progression [14]. The reason for this is that type IIA procollagen can be re-expressed in OA cartilage as a repair mechanism [59]. In contrast, a recent study reported that risk of progression was also associated with low serum levels of PIIANP among patients characterized by mild and moderate knee OA [16]. Therefore, further verification is required. For advanced OA, a previous study of Garnero et al. observed an association of decreased serum levels of PIIANP and progression in patients with medial compartment knee OA [15], reflecting an absence of effective cartilage repair mechanism in advanced OA. Taken together, the value of serum PIIANP needs to be considered carefully when evaluating OA.

Next, researchers have also been focused on the many cleavage fragments from type II collagen that are secreted during cartilage breakdown. One of the most intensively studied fragments is C telopeptide fragment of collagen type-II (CTX-II). The concentration of CTX-II in synovial fluid was reported to be higher in patients with primary knee OA (diagnosed by radiography) than in healthy people. CTX-II also increases in people with an isolated meniscus tear or an isolated anterior cruciate ligament rupture or combined meniscus tear and ligament tear [23], and these marker levels can decrease with effective treatment.

It has also been observed that the CTX-II concentration in urine increases in patients with hip, hand, facet or knee joint OA, and this can be used as a prognostic marker as the CTX-II level correlates with disease score and progression [17,18,22]. Another study by Rotterud et al. showed that patients with a focal cartilage lesion of the knee have higher concentrations of urinary CTX-II than healthy individuals and the CTX-II concentration decreases during rehabilitation [19], suggesting the CTX-II biomarker can be used to monitor treatment effects.

It has been observed that the synovial fluid concentration of C-terminal neopeptide (C2C), another fragment derived from type II collagen degradation, is higher in patients with injured knees from 0 days to 7 years after injury than in healthy people [25]. According to Conrozier et al., serum C2C correlates with joint space narrowing (JSN) in patients with unilateral hip OA [24], and this may be a prognostic marker for patients with isolated hip OA. Urine C2C has been suggested as a diagnosis marker of knee OA because C2C levels are higher in OA patients than in controls [26].

In addition, it was reported that patients with mild or severe knee OA have a higher serum concentration of CIIM than people with no OA [27]. In a study of hand OA, Punzi et al. found elevation of Coll2-1NO2, a nitrated form of type II collagen-derived fragment, in the serum of patients with erosive hand OA compared to levels in non-OA patients [29]. It has been indicated that the average measurement of urinary HELIX-II peptide in patients with knee OA is higher than that in normal controls [28].

In addition to type II collagen, several recent studies have investigated potential markers that come from type III and type X collagen [30,31]. OA is characterized by the changing of the chondrocyte phenotype into one of hypertrophy [2] and increased expression of collagen type X is a hallmark of this change. A study by He et al. showed that the serum level of C-terminus of collagen type X (C-Col10) is higher in patients with a Kellgren–Lawrence (KL) score 2 classified by radiography compared to patients with a KL 0 [31]. This study also found that C-Col10 correlates with serum C2M and C-reactive protein (CRP), an inflammatory marker, suggesting a prognostic marker for inflammatory OA.

After collagen type II, aggrecan is the second most abundant protein in the cartilage matrix. Epitope 846 concentration (an indicator for aggrecan synthesis) in joint fluid was elevated in primary OA patients and patients with knee injury versus healthy controls [32] and was highest in patients with primary OA. ARGS, fragments cleft from aggrecan by aggrecanase, has been shown to increase in knee OA and after knee injury (from 0 to 12 weeks) [33]. Moreover, synovial fluid (SF) ARGS neoepitope concentrations correlated with the Western Ontario and McMaster Universities (WOMAC) stiffness scores in OA patients undergoing total knee replacement as shown in a study by Germaschewski et al., suggesting an early marker for OA therapeutics [34].

Several other markers derived from non-collagen and non-aggrecan proteins have also been identified (e.g., cartilage oligomeric matrix protein—COMP; pentosidine) (see Table 1). COMP is a non-collagen protein related to the thrombospodin family, and also a constituent of articular cartilage. A study by Fernandes et al. suggests COMP as a diagnostic marker for early OA as they found serum COMP level increases in patients with the symptom of pain in the knees and without any radiological abnormalities, indicating early cartilage damage in these patients compared with healthy controls [35]. A later study by Verma and Dalal supports this idea with a similar finding that the serum level of COMP correlates with pain score but not radiological grading and that it also negatively correlates with disease progression. Thus, COMP levels can also be used as a prognostic marker to predict patients at risk of rapid progression [36]. Pentosidine, an advanced glycation end-product has been shown to increase in serum and synovial fluid of OA knee patients compared with normal people [37] and patients with higher baseline serum levels of pentosidine seem to have faster radiological progression (determined by JSN) as observed over a 2-year period, suggesting a prognostic marker of pentosidine for knee OA [38].

In addition to COMP and pentosidine, fibulin-3 peptides (Fib3-1 and Fib3-2) and follistatin-like protein 1 (FSTL1) have been recently identified. FSTL1 is a secreted glycoprotein that has been implicated in arthritis [60]. Elevated serum and SF levels of FSTL1 in knee and hip OA patients were reported by Wang et al. It was also observed that serum FSTL1 levels were elevated in females compared to males among both in controls and patients. Furthermore, serum FSTL1 in female patients significantly correlates with KL grade, JSN and WOMAC stiffness, suggesting a prognostic marker for measurement of the severity of OA [39]. While the role of fibulin-3 in osteoarthritis is still unknown, a recent study by Henrotin et al. suggests that Fib3-1 and Fib3-2 are potential diagnostic biomarkers of OA, demonstrating that serum levels of Fib3-1 and Fib3-2 in severe knee OA patients are higher than those in healthy controls [40]. Moreover, investigating middle-aged, overweight and obese women, Runhaar et al. indicated that Fib3-1 correlates with the incidence of radiographic knee OA defined by American College of Rheumatology (ACR) criteria and chronic pain at 30 months of follow-up [41].

Proteolytic enzymes such as matrix metalloproteinases (MMPs) and aggrecanases (specifically, disintergrin and metalloproteinase with thrombospondin-like motifs 4 and 5, ADAMTS-4, 5) are directly responsible for the degradation of cartilage matrix molecules [61] and have attracted researchers attempting to identify these enzymes as OA biochemical markers. It has been shown that MMPs such as MMP-1, MMP-3, MMP-9 and MMP-13 have important roles in cartilage matrix degradation in OA joints [62,63]. A study by Li et al. showed that the concentration of MMP-3 and MMP-9 in plasma increased in patients with knee OA, and these levels correlated with the severity of clinical symptoms (evaluated by Lequesne’s index). Therefore, this study suggests that these markers can be used to diagnose early OA [42]. In addition, the MMP-1 level in SF tends to decrease in patients with severe OA compared to that in patients with no or moderate OA (evaluated by grading). The reason for this is that MMP-1 is expressed mainly in superficial cartilage zones that are deteriorated during the progression of OA [43]. The SF level of MMP-13 has been studied and found to correlate with OA grade and postoperative WOMAC scores [44]. Moreover, ADAMTS-4 has been suggested as a possible diagnostic marker as its levels are significant higher in intermediate and advanced OA patients than in controls [45].

While MMP and ADAMTS levels reflect degradation of articular cartilage, their inhibitors may reflect synthesis of cartilage. It has been shown that the concentration of tissue inhibitor of metalloproteinases-1 (TIMP-1) correlates with that of the 846 epitope and the tissue inhibitor of metalloproteinases-2 (TIMP-2) level correlates with PIICP level (the two indicators for cartilage synthesis) [46].

### 2.2. Markers of Bone Metabolism

Collagen I is one of the main components of the subchondral bone [64]. It has been proven that type I collagen has a role in the progression of OA [65]. Similar to type II collagen, propeptides such as procollagen type I N-terminal propeptide (PINP) and procollagen type I N-terminal propeptide (PICP) are cut off and released during collagen I synthesis and maturation, and the level of these peptides in biological fluids may reflect type I collagen synthesis. Recently, a study by Kumm et al. conducted separate radiographic assessments of JSN and osteophytes among patients with early-stage, progressive knee OA to investigate the relationship between bone markers and different forms of JSN and osteophyte progression. It was found that baseline serum levels of PINP correlate with subsequent tibiofemoral (TF) and patellofemoral (PF) progressive osteophytosis but not with JSN, suggesting PINP as a prognostic marker for osteophyte progression [47].

Osteocalcin (OC) is a non-collagenous bone matrix protein, traditionally known as a bone formation indicator. However, a study has shown that OC and its fragments are released from bone matrix during bone resorption, suggesting it is also an indicator of bone turnover [66]. A study by Kumm et al. showed that serum OC is of diagnostic value in knee OA with progression of isolated TF osteophytes and knee OA with progression in the TF alone (TF osteophytes and/or TF JSN) [47]. Additionally, in this study, urinary mid-fragments of osteocalcin (MidOC) were suggested as a risk predictor in OA with progression in TF, TF-isolated osteophytes and TF and PF osteophytes, suggesting urinary MidOC for use as a diagnostic marker of progressive osteophytosis.

Moreover, several markers derived from type I collagen degradation have also been identified. C-terminal type I collagen telopeptide (CTX-I) and N-terminal type I collagen telopeptide (NTX-I) are considered to be resorption markers and their formation depends on cathepsin K, a degradative enzyme secreted by active osteoclast cells. Urinary NTX-I and CTX-I have been shown to increase in progressive knee OA compared with that found in non-progressive OA and controls [48]. The baseline urinary level of a non-isomerized form of CTX-I epitope called alpha-CTX-I has been shown to be associated with dynamic bone turnover in OA knees and progression of both osteophytes and JSN [49]. Moreover, a transversal study of Kraus et al. showed that both alpha-CTX-I and beta-CTX-I (isomerized form of CTX-I epitope) levels in urine were associated with knee OA progression [16]. Besides, urinary levels of pyridinium cross-links of collagen, pyridinoline (PYD) and deoxypyridinoline (DPD) increase significantly in patients with late stage OA (radiographic score 3 and 4) compared with levels in early OA (radiographic score 1 and 2) [50].

### 2.3. Markers of Synovium Metabolism

Hyaluronic acid (HA) is one of the important molecules produced by synovial lining cells (synoviocytes) and functions in lubrication of articulating cartilage surfaces; therefore, it helps to maintain the integrity of cartilage surfaces in diarthrodial joints [67]. A change of this molecule by cellular metabolism may affect its ability to lubricate articulating cartilage and cause joint deterioration. However, increased HA in serum has usually been observed in OA patients, suggesting it may be an OA marker. A study by Sasaki et al. investigating patients with KL grade 2 OA of the knee, hip, spine, wrist and finger showed that increased serum HA levels are associated with an increased number of OA joints, primarily relating to knee and finger OA [51]. Observing patients with knee OA for a period of 2 years, Pavelka et al. showed that patients with higher basal serum levels of HA are associated with rapid radiological progression of OA [38]. In the same way, serum HA levels increase in patients with erosive hand OA compared with that in non-erosive hand OA patients, and this marker may help to predict further radiographic progression of OA [52]. In addition, serum HA is considered as a burden of disease markers for patients with severe knee OA (KL 4) as shown by Kaneko et al. [53].

Another molecule, YKL-40, is a 40 kDa glycoprotein secreted by synoviocytes and chondrocytes [68,69]. YKL-40 has been known to increase proteoglycan synthesis [70]. Investigating patients with symptomatic hip OA, a study by Conrozier et al. showed that serum YKL-40 levels increase in patients with OA compared to levels in healthy controls and correlate with serum CRP, an inflammation marker, suggesting that YKL-40 is a marker for OA joint inflammation [54]. In patients with total knee replacement surgery, levels of YKL-40 correlate with MMP-1, MMP-3, interleukin (IL)-6 and IL-17 in SF [55]. Moreover, YKL-40 levels in SF correlate with symptomatic severity determined by WOMAC in patients with knee OA [56].

Glucosyl-galactosyl pyridinoline (Glc-Gal-PYD), a glycosylated analogue of PYD, is released during degradation of synovium tissue [71]. Urinary Glc-Gal-PYD levels have significant increases in patients with knee OA compared to control levels and this marker correlates with WOMAC, suggesting a predictor of pain and physical function [58]. A study on knee OA in men also showed that urinary Glc-Gal-PYD is associated with severity of disease determined by KL-grade, JSN and osteophyte score [57].

## 3. Inflammatory Markers

Previously, OA was traditionally considered a non-inflammation disease. Now, it has come to be appreciated that inflammation relates to OA. The proof that symptoms such as joint pain, swelling and stiffness frequently occur in OA patients clearly reflects local inflammation [72] and increasing evidence shows that synovitis is common in OA joints [73,74]. Moreover, many inflammatory factors, such as cytokines produced by articular tissues, have been implicated in disease pathogenesis [75,76]. Over the years, researchers have made great efforts to identify inflammatory markers associated with OA. Inflammatory markers can be divided into several groups as classified in Table 2.

### 3.1. Bone-, Cartilage- and Synovium-Derived Markers

#### 3.1.1. Cytokines

IL-1β and tumor necrosis factor-α (TNF-α) are predominant pro-inflammatory cytokines and regulate the production of a variety of other pro-inflammatory cytokines, such as IL-6 and IL-8, for the initiation of inflammation cascades [98,99]. These cytokines also function as catabolic factors and have a role in cartilage destruction and progression of OA via activation of proteinases (MMPs and aggrecanases) [100,101]. Investigating patients with grade 3 and grade 4 knee OA, Ozler et al. showed that the serum level of TNF-α correlates with OA grades, with grade 4 serum levels being higher than grade 3 levels [44]. Similar results were reported by Stannus et al., who conducted a longitudinal study of patients with knee OA in which they found that the baseline serum level of TNF-α is associated with JSN and knee cartilage loss [78]. Moreover, soluble TNF receptors (TNF-Rs) in serum from older obese patients with knee OA show a positive correlation with pain, joint stiffness and radiographic severity [79]. For IL-1, it has been demonstrated that the level of a natural antagonist of interleukin-1 (IL-1Ra) in plasma is associated with the severity and progression of symptomatic knee OA as evaluated by JSN, suggesting IL-1Ra as a predictive marker for radiographic OA progression [77].

IL-6, a pro-inflammatory cytokine enhanced by TNF-α and IL-1β, has been known to inhibit type II collagen synthesis [102]. A study on hip OA showed that the IL-6 level in serum correlates with JSN in a group of women with OA [80]. The serum level of IL-6 is also associated with pain in early-stage knee OA in women [81]. Moreover, a longitudinal study on women with knee OA through 15 years of follow-up reveals that higher level of serum IL-6 is associated with an increased chance of diagnosis of OA, suggesting IL-6 is a potential marker for early diagnosis of OA [82].

Other pro-inflammatory cytokines that have been suggested as potential markers for OA include IL-15 and IL-18. Serum IL-15 levels are significantly higher in OA patients compared with levels in control patients and positively correlate with pain analyzed by WOMAC scores [83]. Levels of IL-18 in serum and synovial fluid were observed to be higher in knee OA patients than that in healthy controls [84].

In addition, anti-inflammatory cytokines such as IL-2 and IL-4 have been the focus of a recent study in which elevated IL-2 and IL-4 levels were observed in the plasma of knee OA patients. IL-4 was especially correlated with the radiographic severity of the disease [85].

#### 3.1.2. Chemokines and Growth Factors

IL-8, known as an angiogenic chemokine, functions in activating neutrophils. The serum level of IL-8 has been shown to be positively associated with the severity of knee OA, particularly, severe knee OA patients (KL grade IV) have a higher serum IL-8 level than those with KL grade 0 or 1 [86], whereas increased levels of this cytokine were observed in SF of OA patients with knee surgery compared with that found in patients with knee injury [87].

Vascular endothelial growth factor (VEGF), a potent angiogenic factor, plays a role in OA [103]. VEGF in SF has been shown to be positively correlated with OA severity as defined by KL grade [43]. Both plasma and SF VEGF exhibited a positive correlation with radiographic severity [88], suggesting VEGF as a prognostic marker for OA.

#### 3.1.3. Lipid Mediators

Prostaglandin E2 (PGE2) is a main inflammatory mediator in OA and other diseases. Baseline plasma levels of PGE2 and another lipid mediator, 15-hydroxyeicosatetraenoic acid (15-HETE), have been shown to be elevated in patients with symptomatic knee OA versus levels in non-OA controls, suggesting these lipid mediators are useful as diagnostic and prognostic markers [89].

### 3.2. Markers Related to Other Tissues

#### 3.2.1. Acute Phase Protein

C-reactive protein (CRP) is an acute phase protein which is synthesized and released mainly by the hepatocytes after cytokine stimulation [104]. Catabolic rate of CRP in blood was shown to be constant in all conditions of health and disease (half-life 19 h) and circulating CRP depends on its synthesis rate [105]. Therefore, elevated serum CRP reflects the disease activity that stimulates CRP production. Studies show that serum CRP in patients with knee OA is negatively associated with clinical symptoms such as muscle strength [90] and knee pain at night and when sitting or lying [91]. Serum CRP levels were shown to correlate with KL grade, with the most-sensitized group containing more women than men [92]. Moreover, it was observed that serum CRP levels are higher in erosive hand OA patients than in non-erosive OA patients. CRP was shown to correlate with joint count and radiographic score, suggesting that it plays a role as a marker for erosive hand OA activity [93].

In addition to CRP, MMP-dependent degradation of CRP (CRPM), a degradation fragment from CRP formed after CRP has been synthesized and deposited in the joint, was recently reported. It was shown that levels of CRPM in serum were associated with risk of OA progression in patients with knee and hip OA [94].

#### 3.2.2. Obesity-Associated Factors

Adipokines are bioactive substances (peptides or cytokines) which are derived from adipocytes of white adipose tissue and function as pro-inflammatory factors. They are regarded as a contribution to “low-grade inflammatory state” in obesity [106]. The best known adiopkines are leptin, adiponectin and resitin. Adiopokines, which are not only generated from systemic adipose tissues but also from infrapatellar fat pads (local adipose tissues), play an important role in the development and progression of knee OA [107]. Studies show that adipokines can increase production of MMPs [108,109], suggesting that adipokines have a role in cartilage degradation. Higher serum levels of adipokine were observed in patients with severe knee OA compared to controls without radiographic signs of OA [110]. Investigating adioponectin in male OA patients with knee arthroplasty, Koskinen et al. showed that the plasma levels of adiponectin were associated with radiological severity and correlated with plasma levels of COMP and MMP-3 [95]. Additionally, the plasma level of resitin was shown to be associated with the severity of knee OA as defined by KL grade [86]. According to a study by Stannus et al., the leptin level in serum correlates with hip JSN in female patients, and leptin was reported as a mediator for the association between body composition and hip JSN in women [80].

In addition, apolipoprotein A-I (ApoA1) and cholesterol were observed to increase in SF of RA patients, yet decreases in SF of OA patients and serum levels of ApoA1 and total cholesterol (TC) were higher in OA in comparison with RA, psoriatic arthritis and normal control group [96], suggesting these lipid and apolipoprotein factors can be regarded as possible OA markers.

#### 3.2.3. Other Factors

C-C chemokines including CCL2, CCL3, CCL4 and CCL5 are chemotactic chemokines secreted by macrophages and are known to have a role in OA [111,112,113]. Zhao et al. showed that the plasma levels of CCL3 and CCL4 are elevated in patients with X-ray-defined OA compared to pre-X-ray-defined knee degeneration patients (no obvious sign of X-rays but cartilage degeneration was detected by MRI or arthroscopy) and healthy controls. Specially, CCL3 is elevated in pre-X-ray-defined patients and CCL3 has a high ability to discriminate pre-X-ray patients from healthy people, suggesting CCL3 is a potential diagnostic marker for early detection of the disease [86]. Recently, it was reported that CCL2 concentrations in SF are positively correlated with pain score as defined by WOMAC, suggesting that CCL2 is a marker for symptomatic severity of OA [97].

Furthermore, myeloperoxidase which is released by activated neutrophils is known to affect degradation of collagen components of cartilage via regulating oxidant factors [114], so that myeloperoxidase (MPO) is suggested as diagnostic marker for detection of early OA. In the erosive hand OA, increased value of serum MPO may reflex more expression of inflammatory signs. In fact, MPO and other collagen biomarkers were correlated with radiography and clinical severity of the disease, indicating these biomarkers could be promising specific markers of hand OA disease activity [29].

**Figure 2:**
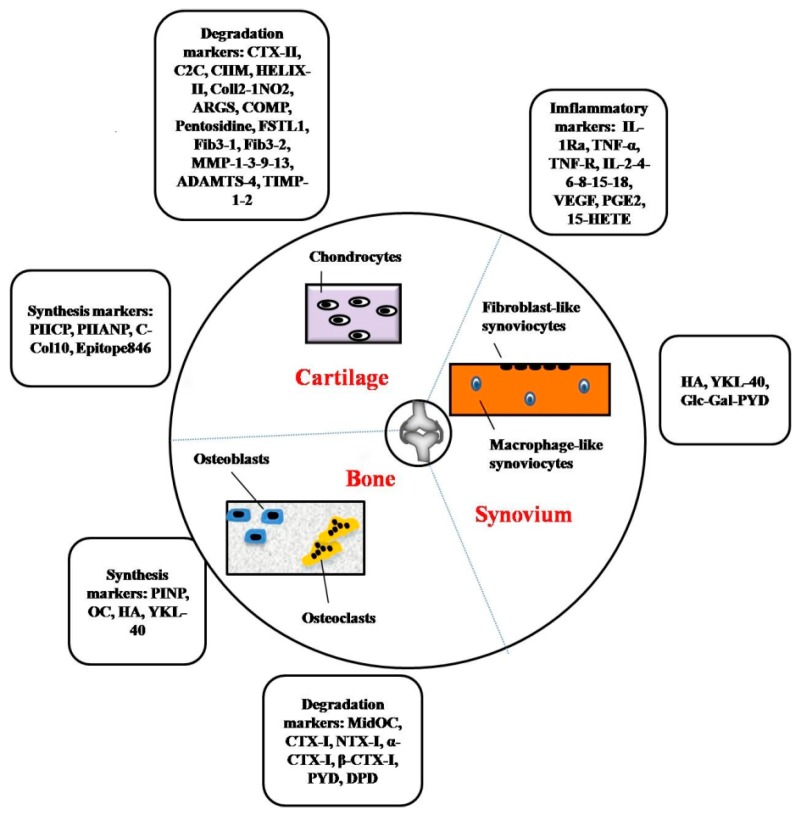
Schematic diagram of cartilage-, bone- and synovium-derived markers in osteoarthritis. Articular cartilage, subchondral bone and synovium are the main sources of many osteoarthritis markers. Generation of these molecular markers is closely related to metabolism of bone, cartilage and synovium via activities of chondrocytes, osteoblasts, osteoclasts and synoviocytes. In addition, inflammatory markers, such as growth factors and cytokines, are derived from the activities of chondrocytes, macrophages and even osteoblasts and osteoclasts.

Biomarkers for OA that are derived from bone, cartilage and synovium are illustrated in Figure 2.

## 4. Genetic Markers

In addition to studies on cartilage, bone, synovium markers and inflammation markers, there are emerging studies on microRNAs (miRNAs) as markers for the diagnosis and prognosis of OA. miRNAs are regulatory factors that regulate gene expression of catabolic factors such as MMPs, aggrecanases and inflammatory factors such as IL-1β and TNF-α, and also regulate genes and pathways relating to pain [115,116,117,118,119,120,121], suggesting their involvement in disease pathogenesis and progression.

The concentration of miR-132 in the plasma has been reported to be significantly reduced in patients with OA compared to plasma levels in controls, thus potentially providing a diagnostic marker [122]. According to a recent study by Borgonio et al., when measuring expression levels among 380 miRNAs in the plasma of patients with primary knee OA, 12 miRNAs were identified as over-expressed in OA patients compared to expression levels in healthy controls, including miR-16, miR-20b, miR-19c, miR-30b, miR-93, miR-126, miR-146a, miR-184, miR-186, miR-195, miR-345 and miR-885-5p [123]. A 5-year longitudinal study in patients with knee and hip joint OA found that three miRNAs (let-7e, miR-454 and miR-885-5p) are associated with severe knee and hip OA. Whereas let-7e and miR-454 were inversely correlated with severe OA, miRNA-885-5p was positively correlated. Among these, let-7e may be a potential predictive marker for severe knee or hip osteoarthritis [124].

In addition to miRNAs, other genetic factors such as small nucleolar RNA (snoRNA) have also been investigated. A study by Zhang et al. conducted with patients 1 year after surgery on the anterior cruciate ligament (ACL) showed increased serum concentrations of snoRNA U48 and U38 in patients with developing cartilage damage compared to levels in patients without developing cartilage damage or healthy controls, suggesting these genetic factors as early diagnostic markers for cartilage damage in patients after ACL injury [125].

Furthermore, genetic features of human leucotype antigen (HLA) have recently been highlighted as it is involved in pathogenesis of OA. HLA-A1 and HLA-B8 haplotypes was found to be associated with hand OA [126]. It was also observed that HLA-DRB1*02 was associated with OA while DR5 was negatively associated with OA [127]. In study of Riyazi et al., HLA-DRB1*02 was suggested as a risk factor for the development of distal interphalangeal OA [128]. Besides, erosive hand OA was suggested to be highly associated with HLA-B38 and HLA-DRB1*07 [129]. In addition to HLA, investigating single nucleotide polymorphisms (SNPs) on the gene encoding IL-1β, the study by Stern showed an association of a SNP on the IL-1β gene and erosive hand OA [130].

## 5. Conclusions and Future Perspectives

Currently, radiographic analysis along with assessment of pain and discomfort are regarded as the hallmark for the initiation of OA. Though a great deal has been done to identify some reliable biomarkers, only a few of these biomarkers have been applied in clinical settings. Joint tissues undergo metabolic changes long before the onset of structural alterations during early OA stages. Various biomarkers from the tissues undergoing these metabolic changes may provide valuable information either for diagnosis or developing new therapeutic alternatives. Recently, a consensus has been reached for screening multiple biomarkers that can be subsequently derived from various tissues undergoing metabolic changes. The collective assessment of the biomarkers associated with different joint tissue types like cartilage, bone and synovium, products of pathological pathways and even genetic factors, will be required for considering a personalized medication protocol for the treatment of OA in the near future. Moreover, as an endpoint representative of the degradative process during OA, biomarkers must be assessed as potential therapeutic candidates for a new drug development regime for OA. Further studies exploring their participatory role in the pharmacodynamics of OA will provide a more credible answer for the feasibility of these biomarkers within clinical settings. Early diagnosis of OA using biomarkers will help physicians to not only develop a strategy for treating OA at early stages but will even prove beneficial in reducing the cost of treatment for patients.

## Figures and Tables

**Table 1:** Selected OA biomarkers of bone, cartilage and synovium metabolism and studies of these markers in patients.

Tissue Origination	Molecule Type Origination	Markers of Synthesis	Markers of Degradation	Sample Type	References
Cartilage	Type II collagen	PIICP ^2^		SF	[11]
		PIIANP ^2^		S	[12,13,14,15,16]
			CTX-II ^1,2,3,4^	U	[17,18,19,20,21,22]
			CTX-II ^2^	SF	[23]
			C2C ^3^	S	[24]
			C2C ^2^	U, SF	[25,26]
			CIIM ^2^	S	[27]
			HELIX-II ^2^	U	[28]
			Coll 2-1 NO2 ^1^	S	[28,29]
	Type X collagen	C-Col10 ^2^		S	[30,31]
	Aggrecan	Epitope 846 ^2^		SF	[32]
			ARGS ^2^	SF	[33,34]
	Non-collagenous and non-agrrecan proteins		COMP ^2^	S	[35,36]
			Pentosidine ^2^	S, SF	[37,38]
			FSTL1 ^2,3^	S, SF	[39]
			Fib3-1 ^2^	S	[40,41]
			Fib3-2 ^2^	S	[40]
	Proteolytic enzymes		MMP-3, -9 ^2^	S	[42]
			MMP-1, -13 ^2^	SF	[43,44]
			ADAMTS-4 ^2^	S	[45]
	Proteolytic enzyme inhibitors		TIMP-1, -2 ^2^	SF	[46]
Bone	Type I collagen	PINP ^2^		S	[47]
	Non-collagenous protein	OC ^2^		S	[47]
			MidOC ^2^	U	[47]
			CTX-I ^2^	U	[48]
			NTX-I ^2^	U	
			Alpha-CTX-I ^2^	U	[16,49]
			Beta-CTX-I ^2^	U	[16]
			PYD ^2,3^	U	[50]
			DPD ^2,3^	U	[50]
Synovium	Non-collagenous proteins	HA ^1,2^		S	[38,51,52,53]
		YKL-40 ^3^		S	[54]
		YKL-40 ^2^		SF	[54,55,56]
	Type III collagen		Glc-Gal-PYD ^2^	U	[57,58]

^1^ Hand, ^2^ Knee, ^3^ Hip, ^4^ Spine. S = serum, U = urine, SF = synovial fluid; PIIANP: procollagen type IIA N-terminal propeptide; CTX-II: C-telopeptide fragment of collagen type-II; C2C: C-terminal neopeptide; CIIM: matrix metalloproteinase-derived fragment of type II collagen; HELIX-II: helical peptide of type II collagen; Coll 2-1 NO2: nitrated form of triple helical region of type II collagen; C-Col10: C-terminus of collagen type X; Epitope 846: aggrecan chondroitin sulfate epitope 846; ARGS: aggrecanase-generated aggrecan fragment with the ARGS neoepitope; COMP: cartilage oligomeric matrix protein; FSTL1: follistatin-like protein 1; Fib3-1: fibulin-3 peptide 1; Fib3-2: fibulin-3 peptide 2; MMP-3, -9: matrix metalloproteinases 3 and 9; MMP-1, -13: matrix metalloproteinases 1 and 13; ADAMTS-4: metalloproteinase with thrombospondin-like motif 4; TIMP-1, -2: tissue inhibitor of matrix metalloproteinase 1 and 2; PINP: procollagen type I N-terminal propeptide; OC: osteocalcin; MidOC: mid-fragments of osteocalcin; CTX-I: C-telopeptide fragment of collagen type-I; NTX-I: N-telopeptide fragment of collagen type-I; Alpha-CTX-I: non-isomerized C-telopeptide of collagen type-I fragment; Beta-CTX-I: isomerized C-telopeptide of collagen type-I fragment; PYD: pyridinoline; DPD: deoxypyridinoline; HA: hyaluronic acid; YKL-40: cartilage glycoprotein 39; Glc-Gal-PYD: glucosyl-galactosyl pyridinoline, PIICP: procollagen type II C-terminal propeptide.

**Table 2:** Classification of inflammatory markers in OA and studies of these markers in patients.

Tissue Origination	Classification of Inflammatory Markers	Biomarkers	Sample Type	References
Cartilage, bone, synovium-deprived markers	Cytokines	IL-1Ra ^2^	S	[77]
		TNF-α ^2^	S	[44,78]
		TNF-Rs ^2^	S	[79]
		IL-6 ^2,3^	S	[80,81,82]
		IL-15 ^2^	S	[83]
		IL-18 ^2^	S	[84]
		IL-2, -4 ^2^	S	[85]
	Chemokines and growth factors	IL-8 ^2^	S, SF	[86,87]
		VEGF ^2^	S, SF	[43,88]
	Lipid mediators	PGE2 ^2^	S	[89]
		15-HETE ^2^	S	[89]
Liver	Acute phase protein	CRP ^1,2^	S	[90,91,92,93]
		CRPM	S	[94]
Adipose tissue	Obesity-related inflammatory factors	Resistin ^2^	S	[86]
		Leptin ^3^	S	[80]
		Adioponectin ^2^	S	[95]
		ApoA1	S	[96]
		TC	S	[96]
Macrophages	Cytokines	CCL3 ^2^	S	[86]
		CCL4 ^2^	S	[86]
Neutrophils	Enzyme	CCL2 ^2^	SF	[97]
		MPO ^1^	S	[29]

^1^ Hand, ^2^ Knee, ^3^ Hip, ^4^ Spine. S = serum, U = urine, SF = synovial fluid; IL-1Ra: interleukin-1 receptor antagonist; TNF-α: tumor necrosis factor α; TNF-Rs: TNF-α receptors; VEGF: vascular endothlial growth factor; PGE2: prostaglandin E2; 15-HETE: 15-hydroxyeicosatetraenoic acid; CRP: C-reactive protein; CRPM: MMP-dependent degradation of CRP; ApoA1: apolipoprotein A-I; TC: total cholesterol; CCL: C-C chemokine ligand; MPO: myeloperoxidase.
